# Nutrient Value of Leaf vs. Seed

**DOI:** 10.3389/fchem.2016.00032

**Published:** 2016-07-21

**Authors:** Marvin Edelman, Monica Colt

**Affiliations:** ^1^Department Plant and Environmental Sciences, Weizmann Institute of ScienceRehovot, Israel; ^2^Hinoman Ltd.Or Yehuda, Israel

**Keywords:** leaf protein, leaf vitamins, leaf minerals, omega 6/3 ratio, *duckweed*

## Abstract

Major differences stand out between edible leaves and seeds in protein quality, vitamin, and mineral concentrations and omega 6/omega 3 fatty acid ratios. Data for seeds (wheat, rice, corn, soy, lentil, chick pea) are compared with corresponding data for edible green leaves (kale, spinach, broccoli, duckweed). An x/y representation of data for lysine and methionine content highlights the group differences between grains, pulses, leafy vegetables, and animal foods. Leaves come out with flying colors in all these comparisons. The perspective ends with a discussion on “So why do we eat mainly seeds?”

## Leaf vs. seed protein

There is a significant difference between seed protein and leaf protein. Seeds (grains and legume pulses) are in the business of plant reproduction and nurturing the developing plant. Leaves, on the other hand, deal mainly with photosynthesis in the mature plant, a process of harnessing visible radiance to produce carbohydrates, and biochemical energy.

Seed protein is a composite of hundreds of different enzymes and structural proteins (Yang et al., 2013), however, its protein complement is dominated by a family of storage proteins: In corn kernels its zein, which comprises up to 60% of the endosperm protein (Larkins and Holding, 2009); in wheat grains its glutenins, which accounts for 40% of the grain protein (Liu et al., 2012); in the rice grain its glutelins, which comprise over 80% of the seed protein (Shyur et al., 1988).

**Figure 1:**
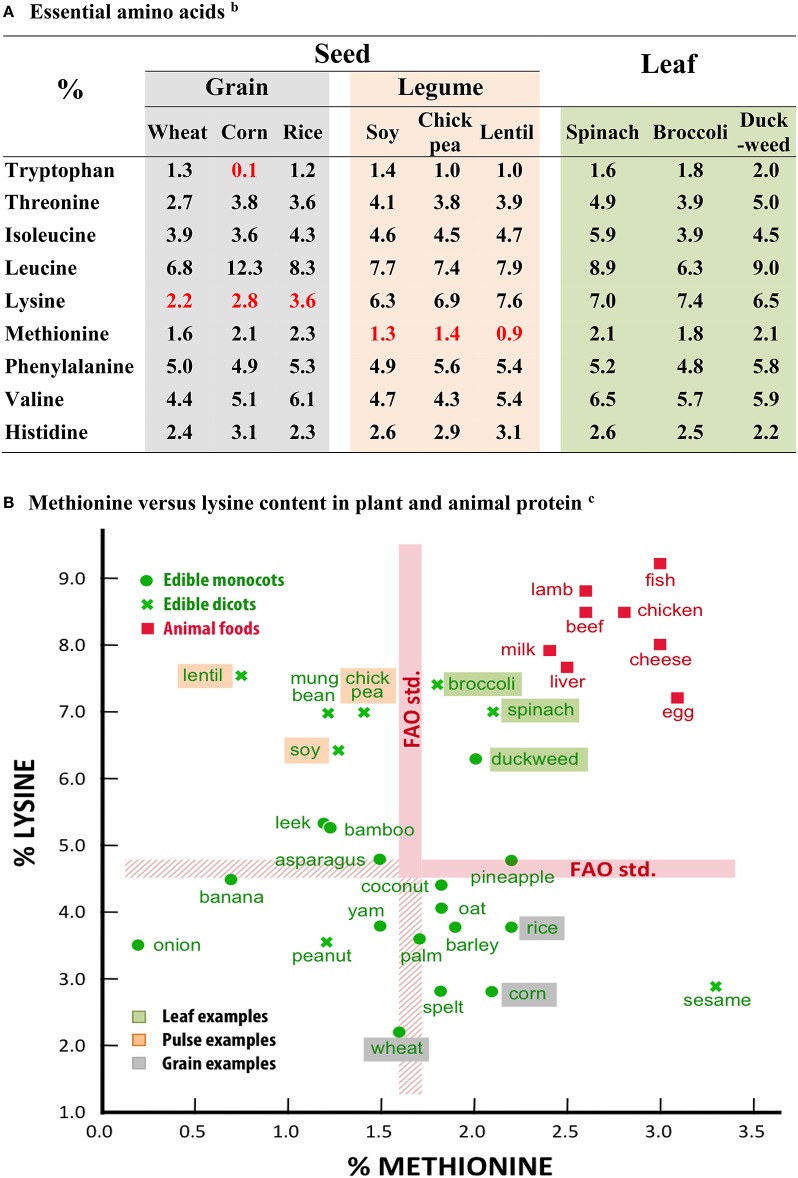
**Essential amino acids compositions for some seed and leafy plants^**a**^**. **(A)** Essential amino acids^b^. **(B)** Methionine vs. lysine content in plant and animal protein^c^. ^a^Values calculated as percent of total amino acids. Data for dried duckweed were determined by Eurofins USA for a local Israeli isolate of *Wolffia* sp. Data for all other plants were abstracted from http://nutritiondata.self.com and are based on USDA National Nutrient Database for Standard Reference (http://ndb.nal.usda.gov/ndb/foods). ^b^All values meet WHO/FAO minimal adult indispensable amino acid requirements (WHO technical Report Series 935, 2007), except for values in red. ^c^The thickness of the FAO standard lines is due to different requirements for “adults” and “children and adolescents” (WHO Technical Report Series 935, 2007). The range varies, respectively, from 1.6 to 1.7 for Methionine and 4.5 to 4.8 for Lysine.

Storage protein imparts individuality to the seed grain: The insolubility of zein in water (Shukla and Cheryan, 2001), the elasticity of glutenin in dough (Kieffer, 2006), the gelling of glutelin in rice (Agboola et al., 2005). However, along with individuality, an imbalance in nutritional composition often crops up. Many seeds are deficient in one or more of the essential amino acids that our bodies cannot synthesize and which we obtain solely from food intake. For example, several cereal grains are deficient in lysine and tryptophan, while legume pulses are often deficient in methionine and/or cysteine (Shewry et al., 1995; Figure 1A).

The general difference in amino acid composition among the grains, legumes, and leafy vegetables can readily be visualized by comparing methionine and lysine values (Figure 1B). The grains and most other monocot food plants are generally poor in lysine (see the boxed positions for wheat, corn, and rice), while the dicot legume pulses are often lacking in methionine (see the boxed positions for soy, chickpea, and lentil). Leafy vegetables on the other hand (see boxed positions for spinach, broccoli, and duckweed) edge into the FAO standard quadrant along with the animal foods.

Leaf protein is likewise composed of hundreds of enzymes and is likewise dominated by a single polypeptide complex: RUBISCO (ribulose 1,5-bisphosphate carboxylase/oxygenase), which is a crucial component in the photosynthetic fixation of atmospheric carbon within green plants. RUBISCO (previously known as Fraction 1 protein), is located in leaf chloroplasts and can account for 50% of total leaf cell protein (Kawashima and Wildman, 1970). In some plants, RUBISCO even crystallizes within the leaf due to its high concentration (Willison and Davey, 1976). Many chloroplast proteins, including RUBISCO, are highly conserved at the gene and protein levels (Sane and Amla, 1991). Thus, RUBISCO is pretty much the same protein in all green leafy plants, with only a few amino acids changes from species to species.

Importantly, RUBISCO is rich in the essential amino acids, with usually eight of the designated nine at percentages meeting FAO (Food and Agricultural Organization of the United Nations) nutritional criteria (Kung and Tso, 1978). Leafy plants such as spinach, broccoli, and duckweed (a monocot plant consisting of nothing much more than a single leaf), in fact provide protein containing all the essential amino acids in percentages meeting FAO standards (Figure 1A). In order to achieve a fully nutritional state, seed protein often needs to be a mix of several sources; for example, the famous combination of sesame seeds (tahini) rich in methionine but poor in lysine, with chickpeas (humus), rich in lysine but poor in methionine (Figure 1B).

## Vitamins in leaves and seeds

Vitamins are essential nutrients required in small amounts that our bodies are not able to supply in sufficient quantity. Therefore, they must be obtained from the foods we eat. The complement of vitamins in leaves and seeds are very different. Grains are generally low in vitamins, legume pulses are spotty (for example, green pea is rich in vitamin C but not in other vitamins) while leafy vegetables are often rich in several vitamins. This can be readily seen by comparing vitamin concentrations for green leafy vegetables with comparable data for grains and pulses in USDA's National Nutrient Database (Nutritiondata Tools, 2014). Edible green leaves, including duckweed (Landolt and Kandeler, 1987; Marizvikuru and Gwaze, 2013), generally have at least an order of magnitude more *pro*-vitamin A (i.e., *beta*-carotene), vitamin B1 (thiamine), vitamin C (ascorbic acid), vitamin E (alpha tocopherol), and vitamin K (naphthoquinones) than do grains or pulses (Table 1A).

**Table 1:** **Nutritional compositions for some seed and leafy plants^a^**.

	**Seed**					**Leaf**		
	**Wheat**	**Corn**	**Rice**	**Soy**	**Lentil**	**Kale**	**Spinach**	**Duck-weed**
**A. VITAMIN CONCENTRATIONS**^b^								
Vitamin A, IU (beta-carotene)	9	167	0	114	68	130,000	85,500	77,900
Vitamin B1, mg (thiamine)	0.4	0.2	0.2	0.6	0.6	0.9	0.9	1.1
Vitamin B2, mg (riboflavin)	0.2	0.1	0	1.1	1.3	0.9	1.8	2.8
Vitamin B5, mg (pantothenate)	1	0.5	1.5	1.5	0.4	0.9	0.9	2.1
Vitamin B6, mg (pyridoxal)	0.3	0.3	0.8	0.5	0.5	2.5	1.8	1
Vitamin C, mg (ascorbic acid)	0	0	0	0	2	1014	256	94
Vitamin E, mg (tocopherols)	0.8	0.3	0.2	1.8	–	9.3	18.2	45.7
Vitamin K1, μg (phylloquinone)	1.9	0.2	0	67	–	6900	4400	51
**B. MINERAL CONCENTRATIONS**^b^								
Calcium, mg	34	6.4	10	195	34	846	1036	607
Iron, mg	3.8	2.2	0.4	6	6.4	8.3	28.4	25.7
Magnesium, mg	120	85	36	407	46	265	827	231
Phosphorus, mg	332	250	100	469	276	519	513	1741
Potassium, mg	405	289	78	2387	664	2769	5840	5319
Sodium, mg	3.1	4.6	0	12.3	5.9	214	827	132
Zinc, mg	3	1.6	0.8	3.7	3.2	3.2	5.5	15
**C. OMEGA-6/OMEGA-3 FATTY ACID RATIO**								
ω-6/ω-3	19.4	32.2	4.7	7.5	3.7	0.8	0.2	0.3

a *Derived from the USDA National Nutrient Database (http://nutritiondata.self.com) for: Wheat flour, whole grain; corn flour, whole grain, yellow; rice flour, white, unenriched; soy flour, full-fat, raw; chick pea, mature seeds, raw; lentil, pink, raw; spinach, raw; broccoli, raw; kale, raw. Data for duckweed determined by Eurofins USA for a local Israeli isolate of dried, raw, Wolffia sp*.

b *Values are per 100 g sample. All samples normalized to 10% moisture. “–“ indicates a missing or incomplete value*.

## Minerals in leaves and seeds

Metal ions are crucial for our body. They frequently serve as cofactors in enzymatic reactions and are also important for maintaining protein structure. A third of human proteins bind metal ions, with over 10% of enzymes in our body requiring zinc for activity (Azia et al., 2015). The comparative metal ion profile for leaves and seeds is reminiscent of that for vitamins. Grains such as wheat, rice, and corn are relatively low in metal ions, legume pulses such as soy have increased amounts of several minerals, while green leafy vegetables such as kale, spinach, and duckweed (Feedipedia, 2013) are richer in many minerals (Table 1B).

There is a caveat, however, when considering metal ion data. While the amino acid composition (Atanasova, 2008) and the vitamin profile (Mozafar, 1993) of edible plants can be somewhat influenced by the fertility of the soil or the water in which they are growing, the metal ion composition is often more responsive (Macnair, 2003; Chibuike and Obiora, 2014). Water plants such as duckweed are particularly responsive to metal concentrations in their nutrient medium (Wang, 1990). The upshot is, metal ion concentrations quoted for leaves and seeds are, to a large extent, specific for the conditions of fertilization.

## Omega-6 vs. omega-3 fatty acids in leaves and seeds

Current research indicates that an excess of omega-6 fatty acids in our diets can promote prothrombotic and proaggregatory activity, while omega-3 fatty acids promote an anti-inflammatory and anti-thrombotic physiology (Simopoulos, 2002, 2006). There is, in general (with exceptions, such as chia seeds (Nutritiondata Products, 2014), a stark difference between seed and leaf fatty acid compositions. While the former are high in omega-6, the latter are high in omega-3 (Table 1C). In addition, α-linolenic acid, which is abundant in many green leafy vegetables and is a major source of omega-3, can metabolize in our bodies to longer chain fatty acids such as eicosapentaenoic acid, and docosahexaenoic acid. These in turn may beneficially affect chronic disease control (Simopoulos, 2002).

## So why do we eat mainly seeds?

The major portion of the calories in Western and many other diets comes from seeds and seed products, particularly from a very narrow field of four sources: wheat, rice, corn, and soy. The recent, huge increase in the use of soy oil, with its biased linoleic acid/α-linolenic acid ratio, has in fact driven a change in the omega-6/omega-3 ratio from ~ 1:1 to ~10–30:1 in the American population (Blasbalg et al., 2011), a change which may impact negatively on several health aspects (Simopoulos, 2002, 2006). Why if the nutrition value is so clearly on the side of leaves do we feed mainly on potentially problematic seeds and seed products?

The answer seems to lie partly with intrinsic biological issues and partly with big business practice. Roughly speaking, wheat and rice grains, corn kernels, and soybeans are harvested at moisture levels between 15 and 25% (see statistics, Nutritiondata Tools, 2014), while fresh, edible green leaves, such as spinach, broccoli, lettuce, and duckweed each have a moisture level of >90% (see statistics, Landolt and Kandeler, 1987; Nutritiondata Tools, 2014). Therefore, to capture equal amounts of solids, one has to consume about four to six times more leaves than seeds, grains, or beans. An additional factor is oxalate, which has anti-nutrient activity and is prevalent in leafy vegetables (Aletor and Adeogun, 1995). However, in this regard, seeds have their own Achilles heel in the form of anti-nutritional allergens (Taylor et al., 2015).

External factors are also at play: Commercial seed crops are adept at production of carbohydrates, oils, and proteins. Increasingly used as feed, they are efficiently transmuted into animal protein and processed food products. Moreover, with massive silo storage, grains function as international commercial commodities (Pollan, 2007). In the case of soy beans, an increased demand for soy protein for industrial production of beef and chicken led to an excess of soy oil as a byproduct, which quickly became a food staple for restaurants, and the fast food industry (Blasbalg et al., 2011).

With a growing awareness of health issues generated by seed dominated diets, and the documented abundance of nutrients in leafy vegetables, a move in the West appears to be developing back to leaf-based foods and, importantly, to an increased variety of plant species decorating our meal plate.

## Author contributions

ME conceived and wrote the article. MC grew and prepared the Wolffia samples for analysis and assisted in the construction of Table 1.

### Conflict of interest statement

The authors declare that the research was conducted in the absence of any commercial or financial relationships that could be construed as a potential conflict of interest. MC is employed by Hinoman Ltd. and ME consults for Hinoman Ltd.
